# Scapular Resting Posture and Scapulohumeral Rhythm Adaptations in Volleyball Players: Implications for Clinical Shoulder Assessment in Athletes

**DOI:** 10.3390/sports11060114

**Published:** 2023-06-08

**Authors:** Augusto Gil Pascoal, Andrea Ribeiro, Jorge Infante

**Affiliations:** 1 Biomechanics and Functional Morphology Laboratory (BFML), Interdisciplinary Centre for the Study of Human Performance (CIPER), Faculdade de Motricidade Humana, Universidade de Lisboa, P-1499-002 Lisboa, Portugal; 2 ISAVE, Instituto Superior de Saúde, Rua Castelo de Almourol 13, P-4720-155 Amares, Portugal; andrea.ribeiro@isave.pt; 3 CIR, Escola Superior de Saúde, Politécnico do Porto, Rua Doutor António Bernardino de Almeida 400, P-4200-072 Porto, Portugal; 4 Sports Expertise Laboratory (LPD), Faculdade de Motricidade Humana, Universidade de Lisboa, P-1499-002 Lisboa, Portugal; jinfante@fmh.ulisboa.pt

**Keywords:** shoulder, kinematics, volleyball, functional assessment, sport-related adaptations

## Abstract

Volleyball players develop shoulder sports-related adaptations due to repetitive overhead motions. It is essential to differentiate between these sports-related adaptations and pathological patterns in clinical assessments, particularly on scapular resting posture and scapulohumeral rhythm. Using an electromagnetic tracking system, the 3D shoulder kinematics of 30 male elite asymptomatic volleyball players and a matching control group were recorded at rest and in eight humeral elevation positions, in 15-degree increments from 15 to 120 degrees. The results indicated that the dominant scapular resting posture of the volleyball group was more anteriorly tilted than the control group (Volleyball: mean = −12.02°, STD = 4.16°; Control: mean = −7.45°, STD = 5.42°; Mean difference = 4.57°; STD = 6.85°; CI95% = 2.1° to 7.1°). The scapulohumeral rhythm in the volleyball group showed greater scapular internal rotation (Volleyball: mean = 41.60°, STD = 9.14°; Control: mean = 35.60°, STD = 6.03°; mean difference = 6.02°, STD = 1.47°; CI95% = 4.80° to 7.25°) and anterior tilt (Volleyball: mean = −9.10°, STD = 5.87°; mean = −2.3°, STD = 9.18°; mean difference = 6.88°, STD = 0.66°; CI95% = 6.34° to 7.43°). These findings suggest that volleyball players have developed a sports-related scapular adaptive pattern. This information may be valuable for clinical assessment and rehabilitation planning in injured volleyball players and may aid in the decision-making process for determining a safe return-to-play after a shoulder injury.

## 1. Introduction

Volleyball is a high-intensity sport that demands rapid and forceful movements, especially from the upper extremities. Both indoor and beach volleyball requires players to execute a variety of skills, such as spiking, setting, serving, and blocking, that involve continuous overhead activity and repetitive hitting movements. Elite volleyball players practice and compete for 16 to 20 h per week, resulting in more than 40,000 spikes in a single season [1]. A volleyball spike, also known as a “hit” or “attack”, is a way of scoring a point in volleyball, wherein a player jumps and forcefully hits the ball over the net and into the opponent’s court to make it difficult for the opponent to return the ball. Spikes are high-speed arm movements that require a large range of motion in the upper arm while airborne to strike the ball with maximum force (ball speeds can approach 28 m per second) [2]. During a spike, the shoulder joint reaches 145 degrees of elevation, 75 to 80 degrees of horizontal abduction, and 115 degrees of external rotation [3]. These actions result in considerable stress on the muscles and joints of the upper body, making volleyball players vulnerable to shoulder injuries [4,5], like other overhead athletes such as throwers, swimmers, and tennis players. Therefore, understanding the shoulder complex’s biomechanics, particularly those of the scapula, is essential for preventing and managing shoulder injuries in volleyball players. In particular, the scapular resting posture and scapulohumeral rhythm are critical components of shoulder function that have been linked to shoulder injuries in overhead athletes.

The scapular resting posture or static scapular posture refers to the normal, relaxed position of the scapula when the arm is at rest by the side of the body. In turn, the scapulohumeral rhythm can be defined as the coordinated movement between the humerus and the shoulder girdle, scapula, and clavicle during arm motion, particularly arm elevation. This motion pattern is essential for the efficient transfer of force from the torso to the arm during overhead activities [6]. Disruptions to the scapulohumeral rhythm, also known as scapular dyskinesis [7], can result in altered shoulder biomechanics, leading to increased stress on the surrounding soft tissues, potentially causing shoulder pain and dysfunction [8]. Scapular dyskinesis can be caused by a variety of factors, including muscle weakness, tightness, imbalances, poor posture, and underlying shoulder pathology [7]. It is commonly observed in the dominant shoulders of overhead athletes, including volleyball players [9,10]. Clinical assessment of scapular dyskinesis involves observing scapular motion and position during dynamic activities, such as arm elevation, and comparing it to normal movement patterns. However, when assessing the shoulder complex of overhead athletes, such as volleyball players, clinicians need to be able to distinguish between sport-related adaptations and pathologic patterns of scapular resting posture and scapulohumeral rhythm. Sport-related adaptations are normal morphofunctional responses to the repetitive demands of overhead activity, whereas pathologic changes are related to complaints or shoulder injury, and negatively impact performance [11]. Many volleyball players exhibit adaptations in their dominant shoulder, particularly in the scapula, including coracoid tightness/pectoral shortening and an asymmetrical side-to-side scapular resting posture [1,12]. However, these changes have not been significantly linked to shoulder complaints, suggesting that they may be specific adaptations to the mechanical demands of overhead sports, as observed in overhead-throwing athletes [6,8,13]. For all these reasons, it remains unclear whether scapular dyskinesis is a risk factor for shoulder injuries or a normal adaptation to the intensive upper limb use required in overhead sports. Differentiating between shoulder sport-related adaptations and pathological changes during shoulder assessment is challenging. This difficulty arises primarily because there is limited information available on what is considered normal or abnormal for scapular resting posture and scapulohumeral rhythm in overhead athletes, especially in volleyball players. This is mainly because most studies have focused on baseball pitchers [6,8,9,10,14]. This knowledge gap has implications for the clinical assessment of these athletes’ shoulders, particularly in identifying scapular dysfunction related to complaints.

Thus, the purpose of this study was to compare scapular resting posture and scapular kinematics during arm elevation (scapulohumeral rhythm) between two groups of healthy male participants: an overhead-throwing athlete group (volleyball players) and a control group. We hypothesized that asymptomatic volleyball players would exhibit sport-related adaptations of their dominant scapula. Ultimately, the study aimed to provide valuable insights into scapular resting posture and the scapulohumeral rhythm motion pattern in volleyball players, to improve clinical assessment and management of scapular dysfunctions in overhead athletes.

## 2. Materials and Methods

### 2.1. Participants

A sample of 60 male, right-handed volunteers, participated in this study, divided into two groups: overhead-throwing athletes (N = 30; Age: mean = 27.4, STD = 4.3 years; Height: mean = 187.6, STD = 8.6 cm; Mass: mean = 87.6, STD = 10.1 Kg) and non-overhead athletes (N = 30; Age: mean = 29.6, STD = 5.9 years; Height: mean = 178.1, STD = 6.8 cm; Mass: mean = 79.6, STD = 14.8 Kg). The athletes’ group included full-time volleyball players with more than 7 years of experience and exposure of more than 10 h/week. The exposure per week was (mean ± STD) 12.3 ± 3.2 h, and they had 11.7 ± 2.4 years of experience in playing volleyball. Athletes were eligible if they were over 18 years of age and were participating without restrictions in training and competition (practice and games) 6 months before testing. The control group included non-overhead athletes, predominantly soccer players (excluding goalkeepers), who underwent a minimum of 2–3 training sessions per week. Potential control group participants were excluded if they had a history of competitive participation or were currently active in overhead sports such as tennis or swimming. The study excluded participants who had experienced shoulder or elbow pain within 6 months prior to the testing, individuals with neck complaints, those who had suffered a traumatic injury to their upper limbs (such as dislocation or subluxation), and those who had undergone previous shoulder surgery. Male participants were exclusively recruited to eliminate any potential gender-related influences on the results. The study adhered to the principles outlined in the Declaration of Helsinki and obtained approval from the Ethics Committee of Faculdade de Motricidade Humana, Universidade de Lisboa (CE1.142012, 14 November 2012). Writeen informed consent was obtained from all subjects involved in the study. 

### 2.2. Sample Size Estimation

An “a priori” power calculation was performed using G*Power 3.1.9.2 software, based on the findings from a study by Ribeiro et al. [15], which examined the scapular resting posture and kinematics in athletes and non-athletes. For the sample size calculation, we employed a 1-tailed hypothesis, an effect size of 0.67, an alpha-error probability of 0.05, a power (1-β error probability) of 0.80, and an allocation ratio (N2/N1) of 1. Based on these parameters, a total sample size of 58 subjects (29 in each group) was determined.

### 2.3. Instrumentation

A six-degrees-of-freedom electromagnetic tracking device (Hardware: “Flock-of-Birds system” by Ascension Technology; Software: Motion Monitor v7.0), was used to record humeral and scapular kinematics. The hardware configuration included an extended-range direct current transmitter and four sensors, operating at a frequency of 100 Hz. The static accuracy, measured at 1.52 m from the transmitter, was reported as up to 7.62 mm RMS (root mean square) for spatial measurements and 0.53 degrees RMS for angular measurements [15]. In a previous study [16], it was found that within a specific measurement area extending 0.91 m to 1.2 m directly in front of the transmitter, the positional error was 0.7 mm, while the orientation error was 0.27°. The accuracy of these measurements in scapular kinematics was further validated through a bone-pin study [17], which employed electromagnetic tracking sensors attached to the skin over the acromion.

A set-up of four sensors was used: the thorax sensor, placed over the spinous process of T1; the scapula sensor, positioned over the broad flat surface of the posterolateral acromion of the dominant scapula; and the humeral sensor, placed over the posterior aspect of the dominant humerus, distal to the triceps muscle belly. The sensors were affixed to the skin using double-sided adhesive disks [18]. Thorax and scapula sensors were wrapped using athletic tape to minimize any skin–receiver movement, while the humeral sensor was secured using a customized cuff. A fourth sensor mounted on a handheld stylus (±6.5 cm) was used to digitize bony landmarks and link the sensors to local anatomical coordinate systems (LCS). This allowed us to calculate segments and joint rotations by combining the LCSs with the sensor motions, following the shoulder ISB standardization protocol [18].

Shoulder bony landmarks were tracked by palpation and then digitized using the stylus. Previous studies have estimated that the error associated with palpation is around 2° [19], and the resulting orientation error of the shoulder bones, resulting from measurement inaccuracy, is less than 2° [15]. During the bony landmarks’ digitalization process, participants assumed a seated position with their eyes fixed forward and their arms positioned alongside their bodies with their palms facing medially.

The anatomical landmarks digitized included the eighth thoracic vertebra, the xiphoid process, the seventh cervical vertebrae, the jugular notch, the Trigonum Spinae Scapulae, i.e., the medial scapular border at which the scapular spine intersects, the inferior scapular angle, the glenohumeral joint center, the medial epicondyle, and the lateral epicondyle [18]. The glenohumeral joint center was estimated using a least-squares algorithm [20], as the point that moves the least with respect to the scapula when the humerus is moved passively through several short arcs.

### 2.4. Data Processing

To establish the scapular and humeral position and orientation with respect to the thorax, local coordinate systems were constructed in the thorax, scapula, and humerus, based on the digitization of the anatomical landmarks on each segment. This was achieved by embedding a local coordinate system in each segment, and subsequently by calculating the scapular and humeral position and orientation with respect to the thorax. Scapular rotations were defined with respect to the thorax using a Y, X′, Z′′ Euler sequence, as described in Wu, van der Helm, Veeger, Makhsous, Van Roy, Anglin, Nagels, Karduna, McQuade, Wang, Werner and Buchholz [18]. The first rotation (sY) around the *y*-axis describes the scapular internal (positive) and external rotation. The second rotation (sX) around the rotated *x*-axis describes upward rotation (positive) and downward rotation (negative), while the third rotation around the scapular *z*-axis (sZ) describes anterior (negative) and posterior (positive) scapular tilts. Specifically, anterior scapular tilting occurs when the inferior angle of the scapula moves away from the thorax.

The humerus kinematics were calculated relative to the thorax using a Y, X′, Y″ Euler sequence, where the first rotation around the humeral *y*-axis describes the “plane of arm elevation”; the second rotation around the *x*-axis describes the “arm elevation angle”; and the third rotation around the moved *y*-axis (axial arm rotation) defines the humeral external rotation (negative) and internal rotation. In this study, only arm elevation angles were considered.

### 2.5. Procedures

After digitalization, participants were instructed to perform three continuous repetitions of bilateral maximum arm elevation in a sitting position, with elbows fully extended and thumbs pointing up while holding a 2 kg dumbbell in each hand. Participants self-selected their elevation plane. To avoid the potential contribution of thorax motion, only the dominant arm was evaluated, even though both upper limbs were elevated. Dominance was determined by asking participants which hand they used to throw or slam a ball.

Humeral elevation/depression motion began with the arm hanging beside the body in a resting position, progressing toward the maximum elevation point that each participant could obtain, followed by returning to the resting position. The arm elevation angle was defined as the angle between the humerus and the thorax coordinate systems, with zero degrees representing the arm hanging vertically downwards and 180 degrees representing full arm elevation in the coronal plane. The maximum arm elevation angle was determined for each repetition and used for subsequent analysis. The mean value of the maximum arm elevation angles across the three repetitions was calculated for each participant.

### 2.6. Data Reduction and Analysis

In this study, we examine the scapular resting posture and scapular motion during the ascending phase of arm elevation (scapulohumeral rhythm) as dependent variables. Scapular resting posture refers to the scapular rotations recorded when the participant is in a resting anatomical position. To evaluate the scapulohumeral rhythm, the scapular motion was described at eight selected humeral elevation positions, ranging from 15° to 120° humerothoracic angles, in 15° increments. On each of these humeral positions, the scapular position was obtained from three repetitions of the ascending phases of continuous motion of arm elevation/depression.

### 2.7. Statistical Analysis

The dependent variables were assessed for normality using the Shapiro–Wilk Test, which confirmed that they met the assumptions of normality. A two-way repeated measures ANOVA test, with interactions, was performed to compare selected humeral angles (within-factor) and groups, i.e., athletes vs. the control group (between-factor). To assess potential differences in demographic variables such as age (years), height (m), and body mass (kg), as well as differences in scapular resting posture between athletes and the control group, an independent-sample t-test was conducted. The homogeneity of variance, assessed using Levene’s test for equality of variances, was assumed. All statistical analyses were performed using a statistical software package (SPSS, version 16) with a significance level set at 0.05.

## 3. Results

The analysis of demographic variables revealed no significant differences (*p* < 0.05) between the volleyball and control groups, including age (Volleyball players: 25.87, STD = 5.47; Control: mean = 27.77, STD = 4.12; Difference: 1.9, 95% CI = 0.61 to 4.41, *p* = 0.261), height (Volleyball players: mean = 1.87, STD = 0.82; Control: mean = 1.84, STD = 0.60; Difference: 0.02, 95% CI = 0.01 to 0.06, *p* = 0.602), and body mass (Volleyball players: mean = 82.96, STD = 8.04; Control: mean = 80.27, STD = 7.21; Difference: 2.69, 95% CI = 1.25 to 6.64, *p* = 0.341). These findings suggest that the two groups were homogeneous in terms of the mean of these variables.

The scapular resting posture analysis revealed a significant difference in spinal tilt rotation between both groups. Specifically, the athletes exhibited a more anteriorly positioned scapula than the non-athletes (t (58) = −3.66; *p* = 0.021; Mean difference = 4.57°; STD = 6.85°; CI95% = 2.1° to 7.1°).

**Figure 1:**
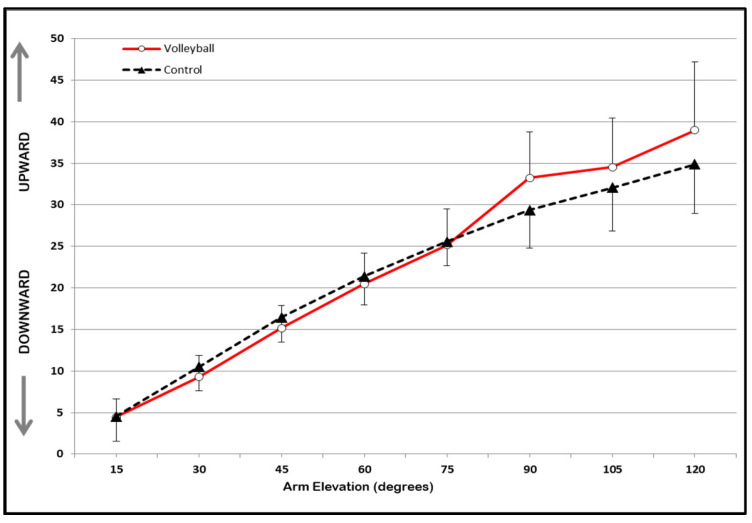
Estimated marginal means of scapular upward/downward rotation (degrees) across the eight selected arm elevation angles in volleyball and control groups. Error bars show STD values.

**Figure 2:**
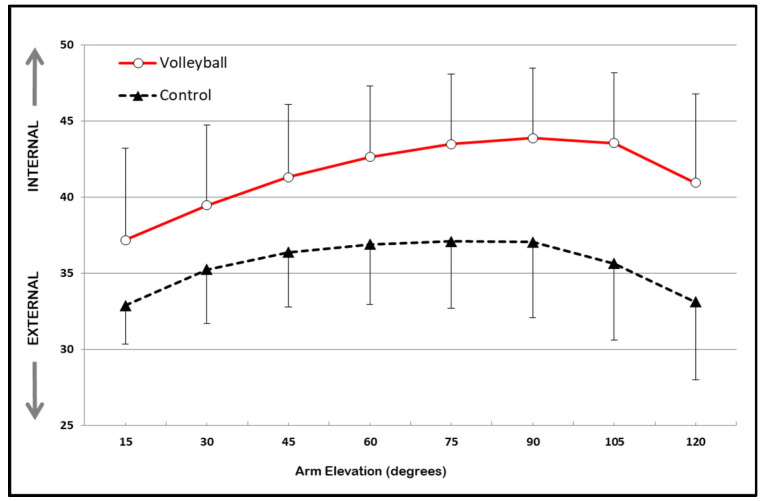
Estimated marginal means of scapular external/internal rotation (degrees) across the eight selected arm elevation angles in volleyball and control groups. Error bars show STD values.

**Figure 3:**
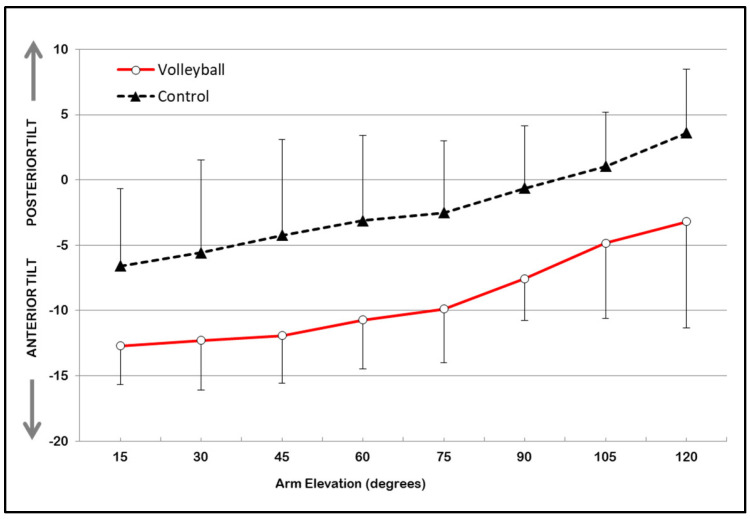
Estimated marginal means of scapular anterior/posterior tilt rotation (degrees) across the eight selected arm elevation angles in volleyball and control groups. Error bars show STD values.

Concerning the scapulohumeral rhythm, Figure 1, Figure 2 and Figure 3 show the estimated marginal means and standard deviation (STD) values for scapular upward/downward rotation, scapular internal/external rotation, and scapular anterior tilt/posterior tilt rotation, respectively. These values are presented for both groups (volleyball and control) across the eight selected humeral elevation angles. The two-way repeated measures ANOVA test, with interaction, revealed no statistically significant interaction effect between groups (volleyball vs. control) or humeral elevation angles for any of the scapular rotations.

The results indicate a significant main effect of the group for scapular internal/external rotation [F (1,58) = 6.79; *p* = 0.012] and scapular tilt [F (1,58) = 27.51; *p* = 0.001]. No significant effect of the group (*p* < 0.05) was found for scapular upward rotation. The volleyball players exhibited significantly greater internal scapular rotation (mean = 41.60°, STD = 9.14°) and anterior scapular tilt (mean = −9.10°, STD = 5.87°) than the control group (internal rotation: mean = 35.60°, STD = 6.03°; anterior tilt: mean = −2.3°, STD = 9.18°), with a difference of 6.02° (STD = 1.47°; CI95% = 4.80° to 7.25°) and 6.88° (STD = 0.66°; CI95% = 6.34° to 7.43°), respectively, across all angles of arm elevation.

## 4. Discussion

### 4.1. Scapular Resting Posture

In this study, asymptomatic volleyball players were found to have a more anteriorly tilted dominant scapula at rest compared to non-athletes (mean difference = 4.57°; STD = 6.85°; CI 95% = 2.1° to 7.1°). These findings are consistent with a previous study by Ribeiro and Pascoal [12], which reported a similar anterior tilt of the dominant scapula in volleyball and handball players compared to non-athletes.

Several studies have compared the dominant and non-dominant scapula to identify asymmetries and patterns of scapular dyskinesia, as well as potential relationships with shoulder complaints [9,10,13,21]. However, limited studies compare scapular resting posture between non-overhead athletes and overhead athletes, specifically volleyball players. Previous studies have reported a side-to-side asymmetrical scapular resting posture in overhead athletes, including baseball players [10,21], handball and volleyball players [12], and tennis players [9,13]. In a study conducted by Oyama, Myers, Wassinger, Daniel Ricci and Lephart [9], 43 overhead athletes, including baseball, tennis, and volleyball players, were found to have a more anteriorly tilted (1.88°) and internally rotated (3.8°) resting posture of their dominant scapula compared to the non-dominant side. Similar results were obtained by Seitz, Reinold, Schneider, Gill and Thigpen [10] in a sample of 45 asymptomatic baseball pitchers, and by Ribeiro, et al. [22] in a sample of volleyball (N = 11), swimmers (N = 11) and handball (N = 11) players.

Previous studies on the resting scapular position of volleyball players have shown conflicting results when comparing the dominant versus non-dominant shoulder. Oyama, Myers, Wassinger, Daniel Ricci and Lephart [9] reported no differences between dominant and non-dominant shoulders in volleyball players, while Ribeiro, Silva, Antunes and Rodrigues [22] found that the dominant scapula in an over-head athlete population was more anteriorly tilted at rest.

### 4.2. Scapular Rhythm during Arm Elevation

In healthy individuals, the scapular motion that accompanies arm elevation, known as scapulohumeral rhythm, is characterized by a pattern of progressive upward rotation, posterior tilt, and either internal or external rotation [23,24]. The results of the present study show that volleyball players have a unique scapulohumeral rhythm in their dominant scapula, which differs from that of non-volleyball athletes. Volleyball players demonstrated greater scapular internal rotation and anterior tilt than non-volleyball athletes. However, there were no significant differences observed in scapular upward rotation. This lack of change in the scapular upward rotation was also observed in a cohort of handball players and swimmers who were investigated for their scapulohumeral rhythm at various degrees of arm elevation (scaption) using a digital inclinometer [22]. Conversely, some studies reveal that professional baseball players [6], tennis players [25], and handball and volleyball players [26] exhibited a greater degree of upward scapular rotation on their dominant scapular compared to a group of non-athletes.

Most recently, Turgut, Colakoglu and Baltaci [25], whilst comparing the scapulohumeral during arm elevation in the scapular plane (scaption) between young tennis players and a control group using an electromagnetic system, found that athletes had greater upward scapular rotation and anterior tilt than the control group, but did not show any significant changes in scapular internal–external rotation. The reasons that explain the differences found in these two studies can be attributed to the instruments used to record scapular movement (inclinometers vs. electromagnetic system), as well as to the specific requirements imposed by different sports.

This study observed a similar increase in scapular internal rotation in volleyball players as compared to professional baseball players who use their dominant shoulder to throw, compared to those who do not engage in throwing activities [6].

Alterations in scapular motion pattern and posture, also called scapular dyskinesis [27], have been previously associated with shoulder pathology but is uncertain whether these alterations represent a cause or effect of the pathology [28,29]. In a systematic review by Burn, et al. [30], scapular dyskinesis was found to have a greater reported prevalence in overhead athletes (60%) compared to the non-athlete population (33%), with volleyball presenting the highest values among the overhead sports (30%). Scapular dyskinesis and rotator cuff dysfunction have been previously associated with shoulder pain in adult athletes [31]. Nevertheless, there is a growing interest in the literature suggesting that scapular motion variability (scapular dyskinesis) in overhead-throwing athletes may simply represent normal kinematic variability. Differences in scapular motion and resting posture found during comparisons side-by-side or between athletes and non-athletes, such as increased or decreased scapular upward rotation, increased internal rotation, and/or variable changes in anterior/posterior tilt, should be assumed to be a sport-related shoulder adaptation in response to intensive and repetitive use of the upper limb.

### 4.3. Shoulder Sport-Related Adaptations in Volleyball

The dominant shoulder of volleyball players, like other overhead athletes, undergoes sports-related biomechanical and morphological adaptations due to repetitive overhead motions such as the serve and spike. These adaptations can lead to changes in range-of-motion (ROM) [32,33] and postural differences when compared to the non-dominant side [33] or non-athletes [12].

These adaptations seen in volleyball players are a result of the high mechanical demands placed on the joint during hitting activities. In fact, during a volleyball spike, elite players perform a circular motion of their hitting arm at high angular velocities, around 900°/s during flexion and 5°/s during internal rotation. The resulting hand speed is approximately 20 m/s, and the ball velocity can exceed 30 m/s [34,35]. The maximum internal rotation torque generates approximately 50 Nm of force, while after ball impact, the shoulder experiences an adduction torque of about 115 Nm and a compressive force of around 800 Nm [36].

Shoulder sport-related adaptations can provide volleyball players with several advantages for performance, particularly during spikes. A volleyball spike is a complex activity that can be divided into three main phases: approach, plant, and jump–flight. The jump phase can be further broken down into three distinct phases: cocking, acceleration, and follow-through [37]. The approach and plant phases play a crucial role in converting the horizontal acceleration of the body into vertical acceleration, which is achieved through an explosive push-off at the end of the plant phase, initiating the jump–flight phase. At the end of the push-off, a force–reaction force generates momentum in the pelvis, which is transferred during the flight phase through trunk rotation and flexion, resulting in rapid shoulder and elbow movements that enable the volleyball player to spike the ball at maximum velocity. During the jump–flight phase, when the feet are not in contact with the ground, the shoulder undergoes a cocking phase that involves a pronounced external arm rotation [37]. This motion incorporates the energy generated during the approach and plant phases to create the mechanical conditions for a powerful acceleration phase that ends with the hand contacting the ball [38]. Thus, the range of motion available for external axial rotation of the shoulder at the glenohumeral joint is critical in determining the amount of energy that can be generated before the impact with the ball.

Volleyball players, similar to other overhead sports athletes, often experience changes in the ROM in their dominant shoulder, specifically affecting the external–internal axial rotation at the glenohumeral joint [38]. This typically involves an increase in the external rotation ROM (“glenohumeral external rotation gain”), with a corresponding decrease in the internal rotation ROM (“glenohumeral internal rotation deficit”), compared to the non-dominant shoulder [39]. This altered ROM on axial arm rotation provides a significant advantage during the acceleration phase of the throwing cycle to generate greater power and velocity for the hand and ball. However, the spike in volleyball demands that the hand contacts the ball at a higher point in the air to avoid being blocked by the opponent, and to attain a better angle for a successful ball attack. This increased ROM in arm elevation requires the involvement of the shoulder girdle and the essential contribution of scapular movement to position the hand at a higher point above the head. The results of our study seem to support this aspect by demonstrating changes in the scapulohumeral rhythm pattern among volleyball players.

The observed ROM adaptations in the dominant scapula of volleyball players can also be attributed to the mechanical stress imposed on the shoulder during the plant phase of the spike. This phase is crucial for converting the body’s horizontal acceleration into vertical acceleration. During this phase, both arms of the volleyball player move progressively from a fully extended position, assumed before the push-off, to a position of arm flexion, while simultaneously the trunk moves into extension and rotation [37]. We believe that this swing pattern of arm motion may have significant implications for scapulohumeral rhythm and the activation of the muscles responsible for scapular motion. These factors could be the genesis for the sport-related adaptations observed in our study, particularly in the scapular resting posture of volleyball players.

Finally, it is important to emphasize that the observed adaptations in volleyball players may increase the risk of shoulder injuries, especially if appropriate training and rehabilitation protocols are not implemented. However, conclusive evidence regarding these patterns and their associations with injuries in volleyball has not yet been established.

### 4.4. Clinical Implications

Our study has significant clinical implications, as it contributes to a normative reference dataset and provides valuable information for the clinical assessment of volleyball players’ shoulders, especially regarding scapulothoracic joint function. Our results demonstrate that asymptomatic volleyball players have normal adaptations in their dominant shoulder, including scapular resting posture and scapulohumeral rhythm pattern, during arm elevation movement. These adaptations should be considered within the confidence interval of the clinical criteria used to define a normal or abnormal function, which guides clinicians in the diagnosis and rehabilitation plan. Such criteria are also crucial in the decision-making process for determining the timing and conditions of an athlete’s return-to-play status.

Verhagen, et al. [40] suggest that shoulder injuries in volleyball can cause an average of 7.9 weeks of training loss and/or competition time, which is significantly longer than other injuries, such as ankle sprains, that cause time loss. This prolonged absence is often due to persistent shoulder pain, especially in extreme ranges of arm movement, and the possibility of injury recurrence. Therefore, clinicians typically advise a more extended rehabilitation period and delay the athlete’s return to activity. However, there is currently insufficient information in the literature regarding functional tests or clinical criteria that can assist clinicians in decision-making, particularly regarding when to recommend a volleyball player return to practice after a shoulder injury.

McCarty, et al. [41] outlined the ideal return-to-play criteria for athletes with shoulder instability, which includes minimal to no pain, subjective satisfaction with the treatment, near-normal range of motion (ROM) and strength, as well as normal functional ability and sport-specific skills. For athletes with scapular dyskinesis, recommendations aim for symmetrical scapular muscle strength, particularly in those who do not perform throwing sports. For those involved in unilateral overhead sports, a target increase of 10% in isometric muscle strength on the dominant side, as measured by a hand-held dynamometer, is recommended [42,43].

Although these recommendations exist, there are no established science-based guidelines for overhead athletes with shoulder dysfunction when it comes to returning to play. While a general decision-based model for return-to-play has been published [44], there is limited information on scapular assessment and the specific guidelines and cut-off values required for the first step of this model (i.e., evaluation of the health status). As a result, clinicians must rely on normative literature data or their own experience and clinical reasoning to determine whether an athlete is ready to return to play.

### 4.5. Limitations

There are several limitations to this study. Firstly, the assessment of scapular kinematics was limited to an arm elevation task and did not involve specific volleyball skills such as serves or spikes. The ballistic nature of these skills could make it challenging to evaluate scapular motion accurately, but it is still essential to explore ways to do so. Secondly, we only assessed the dominant shoulder and did not compare it with the non-dominant shoulder, which could provide valuable insights. Thirdly, we only focused on the arm lift phase and did not assess scapular dyskinesia during the downward phase, which could also be relevant. Finally, we did not previously evaluate scapular dyskinesia, which we believe should be incorporated into clinical reasoning for more effective assessment.

## 5. Conclusions

The findings of this study suggest that the unique demands placed on the dominant shoulder of volleyball players can impact their scapula resting posture and scapulohumeral rhythm pattern, potentially leading to improved upper limb performance. Specifically, volleyball players exhibited a greater degree of anterior tilt and internal rotation in their scapular resting posture when compared to non-athletes. During arm elevation, they also displayed a dominant scapula with more anterior tilt and internal rotation, but no significant differences were observed in scapular upward rotation. These findings are consistent with similar patterns observed in other overhead athletes, such as baseball pitchers, which suggest the presence of a sports-related scapular adaptive pattern that may contribute to enhanced upper limb performance.

## Data Availability

Not available.
